# The Effects of COVID-19 Lockdown on Air Pollutant Concentrations across China: A Google Earth Engine-Based Analysis

**DOI:** 10.3390/ijerph192417056

**Published:** 2022-12-19

**Authors:** Siyu Wang, Haijiao Chu, Changyu Gong, Ping Wang, Fei Wu, Chunhong Zhao

**Affiliations:** 1 School of Geographical Sciences, Key Laboratory of Geographical Processes and Ecological Security of Changbai Mountains, Ministry of Education, Northeast Normal University, Changchun 130024, China; 2 CenNavi Technologies Co., Ltd., Beijing 100094, China; 3 State Key Laboratory of Resources and Environmental Information System, Beijing 100010, China

**Keywords:** urban, remote sensing, air pollutants, COVID-19, Google Earth Engine, China

## Abstract

To overcome the spread of the severe COVID-19 outbreak, various lockdown measures have been taken worldwide. China imposed the strictest home-quarantine measures during the COVID-19 outbreak in the year 2020. This provides a valuable opportunity to study the impact of anthropogenic emission reductions on air quality. Based on the GEE platform and satellite imagery, this study analyzed the changes in the concentrations of NO_2_, O_3_, CO, and SO_2_ in the same season (1 February–1 May) before and after the epidemic control (2019–2021) for 16 typical representative cities of China. The results showed that NO_2_ concentrations significantly decreased by around 20–24% for different types of metropolises, whereas O_3_ increased for most of the studied metropolises, including approximately 7% in megacities and other major cities. Additionally, the concentrations of CO and SO_2_ showed no statistically significant changes during the study intervals. The study also indicated strong variations in air pollutants among different geographic regions. In addition to the methods in this study, it is essential to include the differences in meteorological impact factors in the study to identify future references for air pollution reduction measures.

## 1. Introduction

The COVID-19 outbreak has been one of the most devastating crises throughout the world since World War II. Since the first cases of COVID-19 were reported in Wuhan, China in December 2019, the rapid spread of the virus dramatically challenged people’s lives and their way of living. To limit the virus spread, governments from several countries or regions were forced to take partial or complete lockdown measures, including suspending production and school classes, travel restrictions from the community level to the county level, etc. [1]. These emergency measures significantly reduced industrial and human activities and reduced the emission sources that cause air pollution. Air pollution can induce a variety of fatal diseases, causing great damage to the public’s physical and mental health. Exposure to the air with a high concentration of pollutants can increase the mortality of respiratory and cardiovascular diseases [2,3,4]. Apart from findings related to the COVID-19 lockdown, the role of other environmental governance policies in improving air quality has also been indicated by scholars [5]. For instance, during the 2008 Beijing Olympic Games, the concentration of PM_2.5_ and PM_10_ in Beijing was reduced by 60% [6], and the air quality in Beijing improved significantly during the APEC Summit in 2014 [7]. During the 2010 Shanghai World Expo, the concentration of NO_2_ in the central area of Shanghai, China decreased by 30% [8].

Nitrogen and sulfur dioxides (NO_2_ and SO_2_), carbon monoxide (CO), and ozone (O_3_) are noted as criteria pollutants owing to their adverse impacts on human health and the environment by the United States Environmental Protection Agency [9,10]. As important trace gases in the Earth’s atmosphere, NO_2_ and NO exist in both the troposphere and the stratosphere. NO_2_ and NO enter the Earth’s atmosphere through both natural (wildfires, lightning, and microbiological processes in soils) and anthropogenic processes (notably fossil fuel combustion and biomass burning) [11]. NO_2_ is a reliable indicator for measuring the concentration of nitrogen oxides, since the photochemical cycle between O_3_, NO, and the NO_2_ is in the daytime with a timescale of minutes. CO comes from the combustion of fossil fuels (especially in the northern mid-latitudes), biomass burning, and atmospheric oxidation of methane and other hydrocarbons (especially in the tropics). It can be a major atmospheric pollutant in certain urban areas. Both on-site [12] and the remote-sensing measurements [13,14] indicated the decline of NO_2_ and CO concentration during the lockdowns. Whereas the NO_2_, NO, and CO emissions are closely related to traffic emissions, the formulation mechanism of O_3_ is more complicated. O_3_ in the tropical troposphere reacts with virtually different trace gases, playing various important roles. It can be transported over great distances and affects areas far from the source. Sicard et al. indicated that concentration of O_3_ has increased in many cities around the globe [15]. For SO_2_,the major source is anthropogenic processes. Apart from the impact on the short-term pollution, the formation of sulphate aerosol owing to SO_2_ emission affects the climate through radiative forcing. Bekbulat et al. have indicated that SO_2_ emissions showed an inconsistent global pattern owing to the variations in power generation during the COVID-19 lockdown period [16].

Compared to traditional on-site measurement, satellite-based remote-sensing technology facilitates a deeper understanding of the long-term spatiotemporal patterns of the air quality on a different scale. The Google Earth Engine (GEE) platform has integrated different satellite imagery resources and geospatial datasets all over the world for 40 years and provides a platform with planetary-scale analysis capabilities. With adequate CPU processing power, the GEE makes it easier to analyze and visualize large volumes of data, including Landsat, Sentinel, and MODIS. Though the relationship between the air quality and the COVID-19 measurement has been widely studied based on remote-sensing techniques [12], a comparative and quantitative assessment of this outcome of stringent emission regulation is still needed. Most of the studies on assessing the role the lockdown measures have been carried out for one single city or place [17,18]. The intensity and extent of restrictions caused by COVID-19 vary in different periods. Furthermore, the fluctuations in emissions and meteorological conditions in different cities and places would affect the role of man-made regulations on air pollutant emission.

The main purpose of this study was to analyze the changes in NO_2_, O_3_, SO_2_, and CO concentrations in 16 metropolitan areas with different development routes in China. The different responses of air pollution concentrations to the lockdown in 16 metropolitan areas during three contemporaneous time periods before and after the epidemic lockdown (2019–2021) were analyzed and compared based on the GEE platform. The work contributes to understanding the limitations of man-made emission regulations and provides a guideline for further efficient air quality management and governance.

## 2. Materials and Methods

### 2.1. Study Area

**Figure 1:**
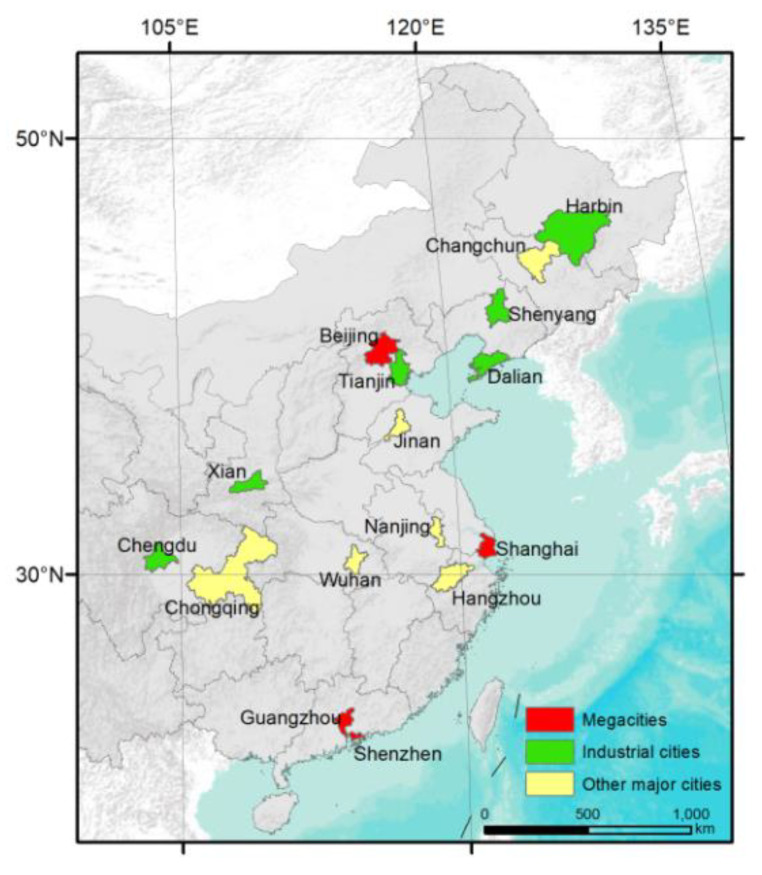
Locations of the 16 selected cities.

We selected 16 metropolises across the mainland of China in this study for analysis (Figure 1). Among them, there are four municipalities directly led by the central government and 12 metropolises that are titled as “sub-province-level division” by the central government for development with priority. We further grouped the areas into three types: megacities (Beijing, Shanghai, Guangzhou, Shenzhen), industrial cities (Harbin, Changchun, Shenyang, Dalian, Tianjin, Xi’an), and major cities (Jinan, Hangzhou, Nanjing, Chengdu, Chongqing). “Megacities” are characterized by high urbanization intensity, with the highest population density and house price. “Industrial cities”, in which the heavy industry bases at the national level are located, have relatively high industrial pollutants emissions. “Major cities” have a relatively high population density and traffic volume. As Wuhan was characterized by the strictest measures and the longest lockdown time during the COVID-19 outbreak, it was analyzed separately in this study. The studied areas are in different latitudinal zones and different economic development zones of China, providing representative cases to study air quality responses to COVID-19 lockdown (Table 1).

### 2.2. Data Sources

The Copernicus Sentinel-5P mission, part of the Copernicus Space Component Program, was launched in 2017 to routinely monitor the atmosphere with a high spatiotemporal resolution. The Tropospheric Monitoring Instrument (TROPOMI) was carried by the Sentinel-5P satellite and is designed to monitor air quality through the measurement of the tropospheric concentration of molecules such as NO_2_, SO_2_, CO, O_3_, etc. With four spectrometers, each electronically split into two bands (2 in UV, 2 in VIS, 2 in NIR, 2 in SWIR), the TROPOMI also provides timely observations of greenhouse gases and other environmental themes including stratospheric ozone conditions and surface UV radiation. TROPOMI products are characterized by a width of swaths of 2600 km and a spatial resolution as high as 7 km × 3.5 km (further refined to 3.5 × 5.5 km^2^ after 6 August 2019). Hence, the products can cover most of the globe daily and have the potential to detect air pollution over individual cities for further comparative assessment among different cities worldwide.

To quantitatively evaluate the atmospheric conditions caused by the COVID-19 epidemic lockdown, the concentration variations of NO_2_, O_3_, SO_2_, and CO were selected as indicators. The TROPOMI NO_2_ processing system is adopted from the retrieval-assimilation-modelling system and established algorithm developments for the DOMINO-2 product and for the EU QA4ECV NO_2_ reprocessed dataset [19]. For the TROPOMI tropospheric O_3_ product, the algorithm was completed by the TROPOMI Level 2 total OZONE and CLOUD products. The algorithm for the TROPOMI SO_2_ product was developed by the Royal Belgian Institute for Space Aeronomy (BIRA-IASB) on the basis of the Differential Optical Absorption Spectroscopy (DOAS) technique [20]. For the TROPOMI CO data processing, the shortwave-infrared CO retrieval (SICOR) algorithm was applied to retrieve the vertical trace gas columns by considering the atmospheric light scattering by clouds. The algorithm also describes the cloud contamination of the measurements with effective parameters [21].

### 2.3. Data Processing

The so-called Level 3 Sentinel-5P data (in the latitude–longitude fixed grid) is provided on GEE. It is based on several product versions of the L2 TROPOMI data (Table 2) and is provided on the GEE. The TROPOMI data for NO_2_, O_3_, SO_2_, and CO were processed using the JavaScript editor within the GEE developer framework. The clouds, surface albedo, snow or ice coverage, signal saturation, and geometry of acquisition are the potential influencing factors for the data quality. Based on the Product User Manual sat the TROPOMI website [22], pixels with quality assurance flags below 75% were removed, and pixels with a “QA value” band less than 0.5 in other datasets (except O_3_ and SO_2_) were removed to ensure data quality. In addition, pixels with a cloud coverage fraction higher than 20% were also removed based on the “cloud fraction” layer of Level 3 Sentinel-5P data provided by the GEE platform. The original measurement unit of the tropospheric vertical column of atmospheric pollution gas is mol/m^2^, which was converted to mmol/m^2^ in this study.

The analysis periods in 2020 were divided into different stages to fully investigate the detailed effects of the lockdown with varying intensity on the atmospheric environment. On 12 January, the World Health Organization (WHO) officially named the novel coronavirus “2019-ncov”. On 30 January 2020, to respond to confirmed cases in each provincial-level administrative region across the country, the central government of China initiated a level I response [23]. On 20 February, although China was facing increasing pressure in COVID-19 prevention, the government still introduced several targeted and coordinated measures to ensure smooth work resumption, especially in sectors of daily necessities. On 17 March, the emergent medical support from various local authorities to Wuhan ended, and the epidemic was brought under control. Since April 28, China began to prevent and control the epidemic in an orderly manner. Therefore, we divided the epidemic lockdown in 2020 into three periods: P1, the early stage of lockdown (from 1 February to 20 February); P2, partial loosening (from 21 February to 17 March); P3, regular prevention and control (from 18 March to 1 May). The atmospheric conditions of the study area in this analytical timeframe in 2020 were compared with those time spans in the “normal” period of 2019 and 2021 (with worldwide regular epidemic prevention).

Non-parametric Wilcoxon tests were used to test whether there was a significant change between different periods. For temporal analysis and comparisons, each pollutant concentration in individual pixels was aggregated into the metropolitan level. The Local Polynomial Regression Fitting (loess) algorithm was applied to indicate the temporal variation in different atmospheric pollutants. All data were processed on GEE and analyzed in R.

## 3. Results

### 3.1. The Effect of COVID-19 Lockdown on NO_2_ Concentration

**Figure 2:**
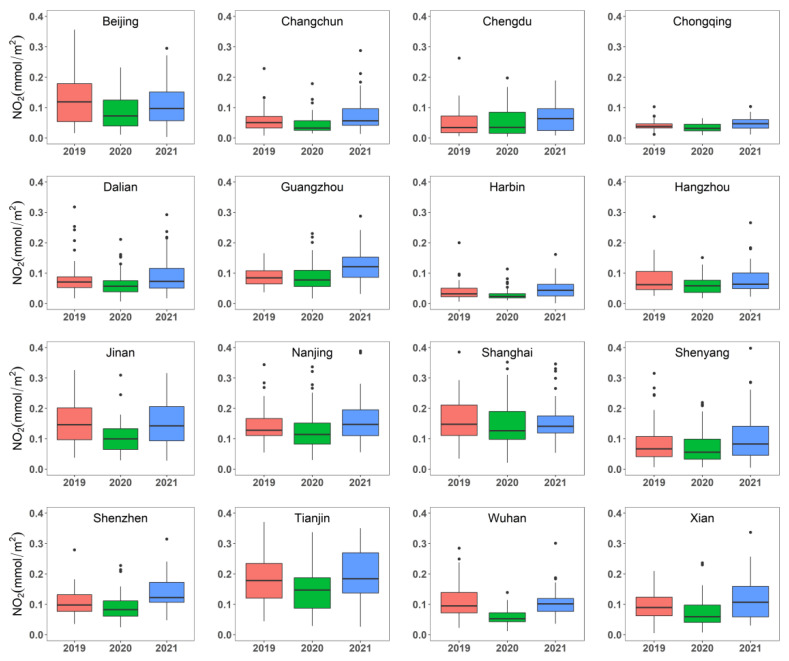
Box plot of NO_2_ concentrations in the study periods for the years 2019, 2020, and 2021.The box represents the inter-quartile range (IQR) and the average value (i.e., the line inside). The two whiskers show (Q3 + 1.5 × IQR) or the maximum value and (Q1 − 1.5 × IQR) or the minimum value, respectively.

Figure 2 shows box plots of NO_2_ concentrations in the 16 selected metropolitan areas for the years 2019, 2020, and 2021 (from 1 February to 1 May). The NO_2_ concentration was highest in Shanghai and Tianjin during the study periods. The NO_2_ concentration in all metropolitan areas decreased dramatically during the lockdown in 2020 compared to the same period in 2019. However, there were apparent differences in NO_2_ concentrations among metropolises within the same city development types, whereas there were no pronounced differences among different groups of metropolises. The concentration of NO_2_ in the metropolitan areas was consistent with the economic conditions and exhibited geographical varying characteristics. The metropolitan areas in Northern China Plain, including Beijing, Tianjin, and Jinan had the highest concentrations of NO_2_ (approximately 0.142 mmol/m^2^ on average), followed by areas in the Yangtze River Delta (i.e., Nanjing, Shanghai, and Hangzhou, approximately 0.129 mmol/m^2^ on average) and the Pearl River Delta (i.e., Guangzhou and Shenzhen). In contrast, the tropospheric NO_2_ concentrations in Northeast China (i.e., Harbin, Changchun, Shenyang, and Dalian) and Western China (i.e., Xi’an, Chengdu, Chongqing, and Wuhan) were significantly lower.

**Figure 3:**
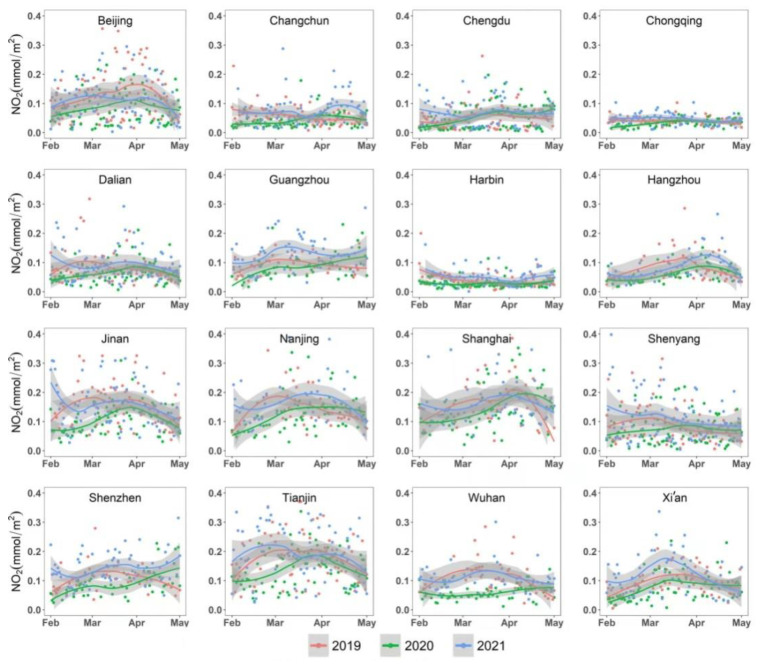
Temporal variation of NO_2_ concentrations in 16 metropolitan areas from 1 February to 1 May in 2019, 2020, and 2021 across the selected lockdown stages. The dots in red, green, and blue colors indicate the values of NO_2_ concentrations in 2019, 2020, and 2021, respectively. The lines and the shaded grey areas refer to the fitted curves modeled by the loess model, with its 95% confidence interval.

Figure 3 shows the time series plots of the tropospheric NO_2_ concentrations in the respective metropolitan areas. Compared to those in 2019 and 2021, the NO_2_ concentrations in the year 2020 showed a relatively high temporal variation across the three stages from April to May. For most places, from the P1 period (i.e., the early stage of lockdown) to the P2 (i.e., the partial loosening period), the concentration sof NO_2_ gradually increased with the gradual relaxation of lockdown measures. The concentrations of NO_2_ in 15 metropolitan areas except Wuhan exhibited a slight upward trend. Though there was no apparent tendency in the NO_2_ concentration after the middle of April, the concentration of NO_2_ showed an upward trend from P1 and P2 to the early stages of P3. In some northern metropolitan areas, such as Beijing, Tianjin, Changchun, Dalian, and Jinan, the concentration of NO_2_ began to decrease from the end of March to the beginning of April. During P3, the concentration of NO_2_ in some areas even exceeded the concentration level in the same period in 2019 (Figure 3). The concentration of NO_2_ in Wuhan also exhibited an upward trend in late April 2019.

According to the results of the non-parametric Wilcoxon test, a significant reduction in NO_2_ concentrations was found over ten cities from 2019 to 2020 (i.e., *p* (2019 and 2020) < 0.05 in Table 3). The most pronounced decrease in NO_2_ was found in Wuhan (−45.1%), followed by those identified as the “major cities” group. Interestingly, the NO_2_ reduction in the industrial cities was comparable to that in the megacities. In 2021, due to the lifting of the lockdown and the nationwide comprehensive work resumption, the emissions of NO_2_ returned to the “base” level. There was no statistically significant change in NO_2_ concentrations between 2019 and 2021 (i.e., *p* (2019 and 2021) > 0.05 in Table 3). Among the 16 cities, the NO_2_ concentration decreased most significantly in Wuhan (45.1%), followed by Jinan (35.1%) and Beijing (34.3%). There was a similar declining tendency in the NO_2_ concentration among all types of metropolises, with an average reduction of 21.22% in megacities, 20.5% in representative cities of heavy industry, and 23.8% in other major cities. Overall, there was an obvious effect of the COVID-19 lockdown on the NO_2_ concentration for most of our study areas.

### 3.2. The Effect of COVID-19 Lockdown on O_3_ Concentration

**Figure 4:**
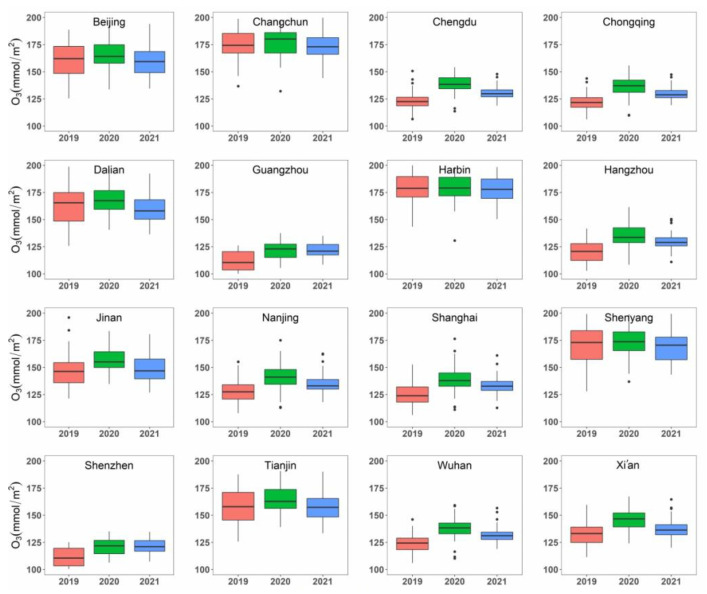
Box plot of the atmospheric O_3_ concentration in the study periods for the years 2019, 2020, and 2021. The box represents the inter-quartile range (IQR) and the average value (i.e., the line inside). The two whiskers show (Q3 + 1.5× IQR) or the maximum value and (Q1 − 1.5 × IQR) or the minimum value, respectively.

There were notable variations in terms of the O_3_ concentrations among the 16 selected metropolises during the study periods (Figure 4). Harbin, Changchun, Shenyang, and Dalian had the highest O_3_ concentration during the study periods (higher than 165 mmol/m^2^ on average), followed by Beijing, Tianjin, and Jinan. For most of the metropolises, there was no considerable variation in O_3_ concentration among the three intervals. Combined with the location of the metropolises (Figure 1), it was indicated that the concentration of O_3_ was partially related with the latitude of the study sites. However, there were no apparent differences among different groups of metropolises. Like the NO_2_ concentration, there were no pronounced differences in O_3_ concentrations among different groups of metropolises.

**Figure 5:**
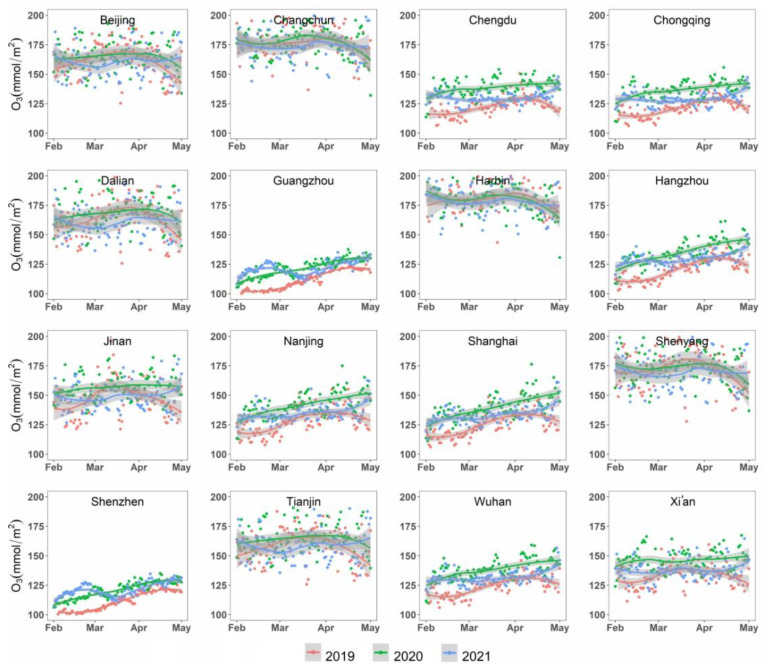
Temporal variation in O_3_ concentrations in 16 metropolitan areas from 1 February to 1 May in 2019, 2020, and 2021 across the selected lockdown stages. The dots in red, green, and blue colors indicate the values of O_3_ concentration in 2019, 2020, and 2021 respectively. The lines and the shaded grey areas refer to the fitted curves modeled by loess, with its 95% confidence interval.

During the intervals in 2019, there were apparent variations in O_3_ concentration, with an increasing and then a decreasing tendency for most of the metropolises. Most turning points occurred in early April, and at the end of March for some northern metropolises (Beijing, Tianjin, Dalian, Jinan, etc.). During the lockdown period in 2020, O_3_ concentrations in 16 metropolises showed a significant upward trend for a relatively long interval. During P3 (regular prevention and control period in 2020), the O_3_ concentrations in six metropolises (Beijing, Tianjin, Changchun, Shenyang, Dalian, and Harbin) showed a downward trend, which is different from the tendency of O_3_ concentrations in other metropolises. Compared to those in 2019 and 2021, the O_3_ concentration in 2020 exhibited a relatively high temporal variation across the three stages. Overall, the volume of O_3_ concentration started to return to the basic level during the study interval in 2021 (Figure 5).

The non-parametric Wilcoxon test further indicated statistically significant changes in the concentration of O_3_ for most of the study areas in 2020 compared to that in 2019. Table 4 indicated that among the 16 metropolises, 12 metropolises showed statistically significant changes (i.e., *p* 2019 and 2020 < 0.05). The O_3_ concentration in Chongqing increased the most (12.4%), followed by Hangzhou (12.0%), Wuhan (11.7%), and Shanghai (11.3%). For megacities and major cities, the O_3_ concentration increased by 7.4% and 7.0% on average, respectively, which was much stronger than that for the heavy industry cities (3.4%). In 2021, there was no statistically significant differences in the O_3_ concentration compared to 2019 (i.e., *p* 2019 and 2021 > 0.05 in Table 4) in Tianjin and Jinan, indicating that these two cities mostly recovered to the pre-epidemic levels of O_3_ concentration. In contrast, the O_3_ concentration in the remaining 10 metropolises decreased slightly in 2021 compared with that in 2020, but the concentration levels were still higher than those in the year 2019 (Table 4).

### 3.3. The Effect of COVID-19 Lockdown on CO and SO_2_ Concentration

Overall, the effect of the COVID-19 lockdown on CO and SO_2_ concentrations was not apparent in most of our study areas. CO and SO_2_ concentrations did not fluctuate significantly before or after COVID-19. According to the results of the non-parametric Wilcoxon test, there were no significant changes in CO concentrations over any type of city from 2019 to 2020 (i.e., *p* (2019 and 2020) > 0.05 in Table 5). The CO concentrations in the megacities (approximately 54.856 mmol/m^2^ on average) were considerably higher than those in the other two groups of cities. In addition, there were notable variations in terms of SO_2_ concentrations from 2019 to 2020 for most of the metropolises, except for Shenzhen and Chengdu. The average atmospheric SO_2_ concentrations in the areas in which heavy industry cities are located were significantly higher than those in the areas noted as “major cities” or “megacities”. A reduction in SO_2_ was also expected during COVID-19. However, unlike previous conclusions, the SO_2_ columns did not change obviously during the blockade period.

## 4. Discussion

### 4.1. Response of Different Air Pollutants to the COVID-19 Lockdown

This study generally indicated the effects of the COVID-19 lockdown on the criteria of air pollutants across China. First, during the lockdown in 2020, NO_2_ levels significantly decreased by 21.2% in megacities, 20.5% in representative metropolises of heavy industry, and 23.8% in other major cities. The decreases in NO_2_ concentrations were similar to the findings based in the continental United States [12] and close to several European cities [24]. The decline in the NO_2_ concentration was largely due to the reduction in traffic and industrial activities during the COVID-19 lockdown, which is the main combustion process of human activities related to NO_2_ emissions [11]. This study did find a considerable difference in the responses of NO_2_ emissions among different geographical regions. One possible reason is that the combustion of fossil fuel and road traffic are the largest contributors to NO_2_ emissions. The North Plain has been the most polluted area in China due to coal consumption for heating and power in the winter, in addition to highly developed industrial production activities. Beijing-Tianjin-Hebei is the region with the greatest traffic pressure in China, and the Yangtze and Pearl River Delta regions represent the highest levels of economic development in China. In addition, the main source of air pollution in the Chinese cities with rapid economic development has recently shifted from coal combustion to a mixture of coal combustion and road traffic with increasing vehicle numbers [25]. The declining tendency of NO_2_ emissions was associated with reduced industrial activities and reduced vehicular traffic from people working remotely and limited domestic travel. Meanwhile, the results showed that the NO_2_ decreased significantly for Wuhan (45.1%), which was much higher than other metropolises. A possible reason for this is that Wuhan had the strictest lockdown measurements due to the severity of the COVID-19 situation.

In contrast, COVID-19 lockdown measures led to an increase in O_3_ concentration in most of our study areas. Between 2019 and 2020, the results showed that the O_3_ increased for most of the studied metropolises, including approximately 7% in megacities and other major cities and 11.7% in Wuhan. The tendency of the O_3_ concentration is consistent with other findings e.g., [16,26]. Nevertheless, we also found differences in terms of the magnitude of changes in O_3_ levels for different types of metropolises. The formation of O_3_ is relatively complex and is affected by solar radiation and other atmospheric pollutants [26]. The study indicated that the variations in the O_3_ concentrations among different types of metropolises were not as strong as those among different geographic regions. Climatic and meteorological conditions also affect the atmospheric environment and contribute to the variability in O_3_ concentration [27]. Volatile organic pollutants (VOCs) and nitrogen oxides (NO_x_) emitted by automobile exhaust and industrial production combine with oxygen to generate O_3_ [15,28]. However, fine particulate matter (PM_2.5_) in the atmosphere will react with hydrogen peroxide (HO_2_) and NO_x_ free radicals, thus reducing the concentration of the O_3_ precursor [29]. During the lockdown, when the emission of air pollutants decreased nationwide, and the concentration of fine particles decreased, the O_3_ generation reaction occurred at a relatively high frequency. Meanwhile, the O_3_ generation reaction is the most intense near the equator. Affected by the large-scale circulation of air in the stratosphere, O_3_ is transported to the middle and high latitudes to accumulate, and the pollution problem of O_3_ in middle and high latitudes will be more serious. In our study, we found that the O_3_ concentration was intense in regions with high latitudes. This may be because meteorological factors have a significant impact on the atmospheric environment, and there are various climatic zones in mainland China with a large north–south span. Furthermore, the concentration of O_3_ is also influenced by the elevation. In the areas with relatively high altitudes, O_3_ concentrations tended to be influenced by meteorological factors at a wider spatial scale and the related transport factors. Meanwhile, we investigated the O_3_ concentrations for the metropolitan areas, and the statistics were aggregated based on the individual metropolitan area instead of the urbanized region.

From the above analysis, we also found that effects of the COVID-19 lockdown on both CO and SO_2_ were not significantly revealed by our analysis. First, CO has a longer atmospheric lifetime than NO_2_, so the impact of emission changes would be less localized than that for NO_2_ [14]. For most of the metropolises, especially for the metropolises in Northern China, SO_2_ remained stable throughout the study stages. A similar finding was also mentioned in the study of Wang et al. [30]. They also pointed out some uncertainties related to the measurement of SO_2_ (i.e., GEE Sentinel-5P OFFL SO_2_ data), especially for highly polluted areas in which the actual pollution status tends to be stronger than that revealed by the investigations with satellite imagery [30]. In addition, industry and coal consumption in power plants are the main emission sources of CO and SO_2_ in China. Heavy industry and coal-fired plants still operated during the COVID-19 lockdown. Hence, CO and SO_2_ should be relatively stable. Additionally, compared to external forces (i.e., the COVID-19 lockdown), SO_2_ reductions are more related to natural or random variability. For instance, the lockdown due to the Asian-Pacific Economic Cooperation (APEC) in 2014 did not cause statistically significant SO_2_ concentration differences over the northern China region [31].

### 4.2. Limitations and Possible Improvements

In this study, we investigated the air quality for the metropolitan areas of China, and the statistics were aggregated based on the individual metropolitan area instead of the urbanized region. However, compared to the urbanized areas, the rural areas have more intensified green cover but less traffic flow and fewer economic activities. Differences in the impact of the lockdown with regard to air quality between urban and suburban areas were also proved by other studies i.e., [13]. The boundaries of the metropolitan areas in this study are based on their administrative divisions, and the percentage of the urbanized cover varies among different types of metropolises. The aggregation of the air pollutants’ emission values from the pixel level to the administrative level would lead to uncertainties in the results. Future studies are needed to illustrate the spatial variations within the administrative level and to check the ways in which the lockdown impacted the air quality in urban and rural communities.

Further, the same period in the year 2019 was used as a base time for comparison to check the effect of the COVID-19 lockdown. However, the condition of the pollutant emissions is also affected by meteorological conditions, and using 2019 as the base level could have lead to biased estimations. It should be noted that the four air pollutants used in this study cannot fully represent the air quality. Other studies have also indicated that the meteorological conditions controlled the spatiotemporal impacts’ variation in the pollutant concentration and the air quality i.e., [32]. To provide reasonable guidelines for alleviating pollutant emissions, the meteorological parameters should be taken into consideration, and a relatively long investigation period is warranted for further studies.

Regarding our methods and analysis, there are other studies that have also applied satellite observational data, especially those from TROPOMI, to measure the changes in air pollutant concentration, including NO_2_, SO_2_, aerosol optical depth (AOD), and CO. For instance, Ghasempour et al. (2021) investigated the spatiotemporal density of TROPOMI-based NO_2_, and SO_2_ products and MODIS-derived AOD from January 2019 to September 2020 (also covering the wave of COVID-19) over Turkey based on the GEE platform [33]. By investigating the major twenty locations of urban area clusters across Pakistan, Ali et al. (2021) observed a reduction in NO_2_ emissions by 40% from coal-based power plants, followed by 30% in major urban areas in 2020 compared to the same period in 2019. Nonetheless, a gradual increase has been observed since 16 April due to relaxations in lockdown implementations. In their study, the TROPOMI data was used to measure the changes in NO_2_, SO_2_, and CO [34]. With TROPOMI data, Miyazaki et al. (2020) estimated a reduction in Chinese NOx emissions reaching 36% during the COVID-19 [35]. Hence, the GEE and remote-sensing imagery are helpful for the spatiotemporal evaluation of air quality, especially before and after the COVID-19 lockdown.

In our study, the spatiotemporal variations in ozone O_3_, one of the secondary pollutants, were also included to study the effects of the COVID-19 lockdown on air pollutant concentrations across China. Different from the primary pollutants (NO_x_, volatile organic compounds, CO, SO_2_), ozone O_3_ is one of the secondary pollutants. The related increase in the concentration of O_3_ ozone depends on the relative amplitude in the change of volatile organic compounds and in NOx emissions. Apart from this, different components of the atmospheric system should be comprehensively investigated. Thus, the role of different processes and air pollutants affecting the level of pollutants during the COVID-19 pandemic period can be assessed.

## 5. Conclusions

In this study, four types of air pollutants’ (NO_2_, O_3_, SO_2_, and CO) concentrations were investigated and analyzed before, during, and after the COVID-19 outbreak in 16 metropolitan areas over China. The GEE platform was applied to acquire Sentinel-5P TROPOMI data and further used for data analysis. Results indicated that NO_2_ concentrations significantly decreased by 21.2% in megacities, by 20.5% in representative metropolises of heavy industry, and by 23.8% in other major cities. In contrast, the results showed that O_3_ has increased for most of the studied metropolises, including approximately 7% in megacities and other major cities. Unlike the tendency of NO_2_ and O_3_ concentrations, a significant change was not observed for CO and SO_2_ concentrations. After the lockdown, the concentrations have had a rebounding tendency to return to the concentration levels in 2019.

The study indicated that the decline in the NO_2_ concentration was largely due to the reduction in traffic and industrial activities during the COVID-19 lockdown. Moreover, the O_3_ concentrations have increased, especially in megacities with serious traffic problems and advanced industrial development. The study indicated the variations in air pollutants among different types of metropolises, whereas different regions also showed different trends in terms of air quality amelioration during the COVID-19 lockdown. The finding of this study could benefit future air quality management at the national level. Further work on formulating policies for coordinated emissions reduction should further consider the city types. Combining the GEE platform and remote-sensing imagery contributes to the identification of air pollution reduction potential. It is also important to investigate and examine the different components of the atmospheric system to identify the various pollutants’ concertation levels during the COVID-19 pandemic period. Further, the method in our study is helpful merely to identify the pollutants’ concentrations, instead of pollutants emissions. Thus, the variations in meteorological impact factors, such as temperature, precipitation, boundary layer physics, cloudiness, as well as the respective multiscale transport processes, should be addressed in order to identify the air pollution reduction potential and provide references for air pollution reduction measures in the future.

## Figures and Tables

**Table 1:** Population and car ownership of the 16 selected cities.

	Population of Permanent Residents (Million)	Possession of Private Vehicles (Million)
Wuhan	12.45	3.67
**Megacities**		
Beijing	21.89	5.08
Shanghai	24.87	3.66
Guangzhou	18.74	2.34
Shenzhen	17.63	2.80
**Industrial cities**		
Harbin	10.01	1.94
Xi’an	12.95	3.61
Tianjin	13.87	2.79
Dalian	7.45	1.57
Shenyang	9.07	2.33
Chengdu	20.93	4.56
**Major cities**		
Jinan	11.94	2.51
Hangzhou	9.07	2.28
Changchun	32.05	1.88
Chongqing	9.32	6.97
Nanjing	12.45	2.16

**Table 2:** TROPOMI sensor Level 3 products from Google Earth Engine (GEE) Cloud Data Archive used in processing the Level 3 GEE products.

Resolution	Product	Band Name (Units)
0.01 arc degrees	GEE Sentinel-5P OFFL NO_2_	tropospheric_NO_2__column_number_density (mol/m^2^)
0.01 arc degrees	GEE Sentinel-5P OFFL O_3_	O_3__column_number_density (mol/m^2^)
0.01 arc degrees	GEE Sentinel-5P OFFL SO_2_	SO_2__column_number_density (mol/m^2^)
0.01 arc degrees	GEE Sentinel-5P OFFL CO	CO_column_number_density (mol/m^2^)

**Table 3:** Summary of NO_2_ concentration amount (mmol/m^2^), temporal changes, and non-parametric Wilcoxon test results among different periods.

	2019	2020	2021	Change from 2019 to 2020 (%)	*p* (2019 and 2020)	*p* (2019 and 2021)
Wuhan	0.108	0.059	0.105	−45.1%	0	0.781
Megacities						
Beijing	0.131	0.086	0.11	−34.3%	0.001	0.267
Shanghai	0.195	0.144	0.17	−26.2%	0.023	0.335
Guangzhou	0.099	0.088	0.125	−11.2%	0.274	0.001
Shenzhen	0.107	0.101	0.137	−5.5%	0.082	0.001
mean	0.133	0.105	0.136	−21.2%		
Industrial cities						
Harbin	0.039	0.028	0.048	−27.3%	0	0.055
Xi’an	0.103	0.075	0.116	−27.0%	0.003	0.306
Tianjin	0.189	0.141	0.198	−25.5%	0.001	0.465
Dalian	0.08	0.064	0.09	−20.5%	0.011	0.429
Shenyang	0.085	0.073	0.113	−13.6%	0.147	0.059
Chengdu	0.049	0.052	0.068	6.0%	0.905	0.004
mean	0.091	0.072	0.105	−20.5%		
Major cities						
Jinan	0.164	0.106	0.154	−35.1%	0	0.522
Hangzhou	0.082	0.062	0.082	−23.7%	0.088	0.796
Changchun	0.056	0.043	0.073	−23.3%	0.001	0.051
Chongqing	0.04	0.034	0.046	−15.0%	0.029	0.04
Nanjing	0.145	0.125	0.16	−13.7%	0.04	0.261
mean	0.097	0.074	0.103	−23.8%		

Notes: *p* (2019 and 2020) indicates the *p*-value of the results of the non-parametric Wilcoxon test to evaluate the significance of the NO_2_ concentration differences between the same periods in 2019 and 2020, and *p* (2019 and 2021) indicates the *p*-value of the non-parametric Wilcoxon test of the NO_2_ concentration differences between the same periods in 2019 and 2021.

**Table 4:** Summary of O_3_ concentration amount (mmol/m^2^), temporal changes, and non-parametric Wilcoxon test results among different periods.

	2019	2020	2021	Change from 2019 to 2020 (%)	*p* (2019 and 2020)	*p* (2019 and 2021)
Wuhan	123.801	138.316	131.620	11.7%	2.56 × 10^−19^	1.17 × 10^−09^
Megacities						
Beijing	161.902	165.026	161.655	1.9%	0.185	0.682
Shanghai	125.348	139.559	133.376	11.3%	2.98 × 10^−14^	6.68 × 10^−08^
Guangzhou	111.315	121.632	121.801	9.3%	8.20 × 10^−13^	1.15 × 10^−12^
Shenzhen	111.000	120.832	121.443	8.9%	2.01 × 10^−12^	4.72 × 10^−13^
Mean	127.391	136.762	134.569	7.4%		
Industrial cities						
Harbin	189.252	182.344	182.487	−3.7%	0.023	0.023
Xi’an	133.127	146.500	137.691	10.1%	6.34 × 10^−15^	0.003
Tianjin	158.855	164.369	159.345	3.5%	0.020	0.868
Dalian	164.119	168.556	163.035	2.7%	0.077	0.477
Shenyang	174.443	173.878	172.544	−0.3%	0.807	0.309
Chengdu	122.671	138.664	130.165	13.0%	1.24 × 10^−22^	7.26 × 10^−12^
Mean	157.078	162.385	157.545	3.4%		
Major cities						
Jinan	146.714	156.866	149.061	6.9%	8.60 × 10^−7^	0.257
Hangzhou	120.615	135.060	129.740	12.0%	2.08 × 10^−16^	8.73 × 10^−11^
Changchun	183.640	179.405	178.212	−2.3%	0.214	0.057
Chongqing	121.532	136.601	129.294	12.4%	5.22 × 10^−22^	3.51 × 10^−12^
Nanjing	127.820	141.681	134.945	10.8%	3.33 × 10^−14^	8.42 × 10^−7^
Mean	140.064	149.923	144.251	7.0%		

Notes: *p* (2019 and 2020) indicates the *p*-value of the results of the non-parametric Wilcoxon test to evaluate the significance of the O_3_ concentration differences between the same periods in 2019 and 2020, and *p* (2019 and 2021) indicates the *p*-value of the non-parametric Wilcoxon test of the O_3_ concentration differences between the same periods in 2019 and 2021.

**Table 5:** Summary of CO and SO_2_ concentration amounts (mmol/m^2^), temporal changes, and non-parametric Wilcoxon Test results among different periods.

	CO				SO_2_			
	2019	2020	2021	*p* (2019 and 2020)	2019	2020	2021	*p* (2019 and 2020)
Wuhan	57.745	58.045	60.134	0.776	0.196	0.175	0.158	0.983
Megacities								
Beijing	50.110	48.582	48.788	0.546	0.363	0.411	0.495	0.422
Shanghai	55.405	55.657	54.169	0.904	0.214	0.205	0.195	0.741
Guangzhou	55.871	58.861	56.953	0.105	0.055	0.007	0.002	0.535
Shenzhen	53.429	56.323	54.655	0.164	0.061	0.058	0.050	0.002
Mean	53.704	54.856	53.641		0.143	0.171	0.185	
Industrial cities								
Harbin	43.418	45.140	44.141	0.413	0.542	0.402	0.383	0.277
Xian	44.402	42.510	43.981	0.229	0.217	0.315	0.268	0.050
Tianjin	56.879	55.119	55.162	0.546	0.379	0.346	0.436	0.611
Dalian	53.090	50.707	53.486	0.860	0.378	0.390	0.457	0.874
Shenyang	49.283	49.473	51.994	0.228	0.488	0.457	0.586	0.771
Chengdu	39.187	41.071	42.808	0.254	0.036	0.104	0.024	0.039
Mean	47.710	47.337	48.595		0.340	0.336	0.359	
Other major cities								
Jinan	56.958	54.500	54.731	0.113	0.321	0.322	0.331	0.389
Hangzhou	53.385	56.011	56.659	0.215	0.140	0.150	0.142	0.423
Changchun	45.254	47.441	46.726	0.529	0.578	0.445	0.585	0.328
Chongqing	47.707	49.509	50.768	0.151	0.102	0.109	0.101	0.343
Nanjing	55.346	58.741	59.606	0.041	0.211	0.284	0.202	0.537
Mean	51.730	53.240	53.698		0.270	0.262	0.272	

Notes: *p* (2019 and 2020) indicates the *p*-value of the results of the non-parametric Wilcoxon test to evaluate the significance of the CO and SO_2_ concentration differences between the same periods in 2019 and 2020.

## Data Availability

Not applicable.
